# A Systematic Approach in the Development of the Morphologically-Directed Raman Spectroscopy Methodology for Characterizing Nasal Suspension Drug Products

**DOI:** 10.1208/s12248-021-00605-w

**Published:** 2021-05-18

**Authors:** Gonçalo Farias, Jagdeep Shur, Robert Price, Elizabeth Bielski, Bryan Newman

**Affiliations:** 1 Department of Pharmacy & Pharmacology, Centre for Therapeutic Innovation, University of Bath, Bath, UK; 2 Nanopharm Ltd, an Aptar Pharma Company, Wales, UK; 3 Office of Research and Standards, Office of Generic Drugs, Center for Drug Evaluation and Research, Food and Drug Administration, Silver Spring, Maryland USA

**Keywords:** bioequivalence, generic nasal sprays, morphology, particle size distribution, Raman microscopy

## Abstract

Demonstrating bioequivalence (BE) of nasal suspension sprays is a challenging task. Analytical tools are required to determine the particle size of the active pharmaceutical ingredient (API) and the structure of a relatively complex formulation. This study investigated the utility of the morphologically-directed Raman spectroscopy (MDRS) method to investigate the particle size distribution (PSD) of nasal suspensions. Dissolution was also investigated as an orthogonal technique. Nasal suspension formulations containing different PSD of mometasone furoate monohydrate (MFM) were manufactured. The PSD of the MFM batches was characterized before formulation manufacture using laser diffraction and automated imaging. Upon formulation manufacture, the droplet size, single actuation content, spray pattern, plume geometry, the API dissolution rate, and the API PSD by MDRS were determined. A systematic approach was utilized to develop a robust method for the analysis of the PSD of MFM in Nasonex® and four test formulations containing the MFM API with different particle size specifications. Although the PSD between distinct techniques cannot be directly compared due to inherent differences between these methodologies, the same trend is observed for three out of the four batches. Dissolution analysis confirmed the trend observed by MDRS in terms of PSD. For suspension-based nasal products, MDRS allows the measurement of API PSD which is critical for BE assessment. This approach has been approved for use *in lieu* of a comparative clinical endpoint BE study [1]. The correlation observed between PSD and dissolution rate extends the use of dissolution as a critical analytical tool demonstrating BE between test and reference products.

**Supplementary Information:**

The online version contains supplementary material available at 10.1208/s12248-021-00605-w.

## INTRODUCTION

To demonstrate bioequivalence (BE) with its reference listed drug (RLD), a generic product must demonstrate an absence of a significant difference in the rate and extent of absorption of the active pharmaceutical ingredient (API) when administered at the same molar dose under similar experimental conditions, either single dose or multiple dose [2]. However, the determination of BE for locally acting drugs has been a long standing challenge in the pharmaceutical industry as the absorption of the API at the local site of action is typically difficult to analyze directly, and there is no guarantee that local drug concentration is at equilibrium with the systemic distribution [3–5]. Complex drug-device combination products such as those seen for nasal suspensions further compound this by being dependent on formulation/device, patient/device, manufacturing, and processing factors. Thus, the US Food and Drug Administration (FDA) relies on cumulative evidence of indirect measures to establish BE.

**Fig. 1:**
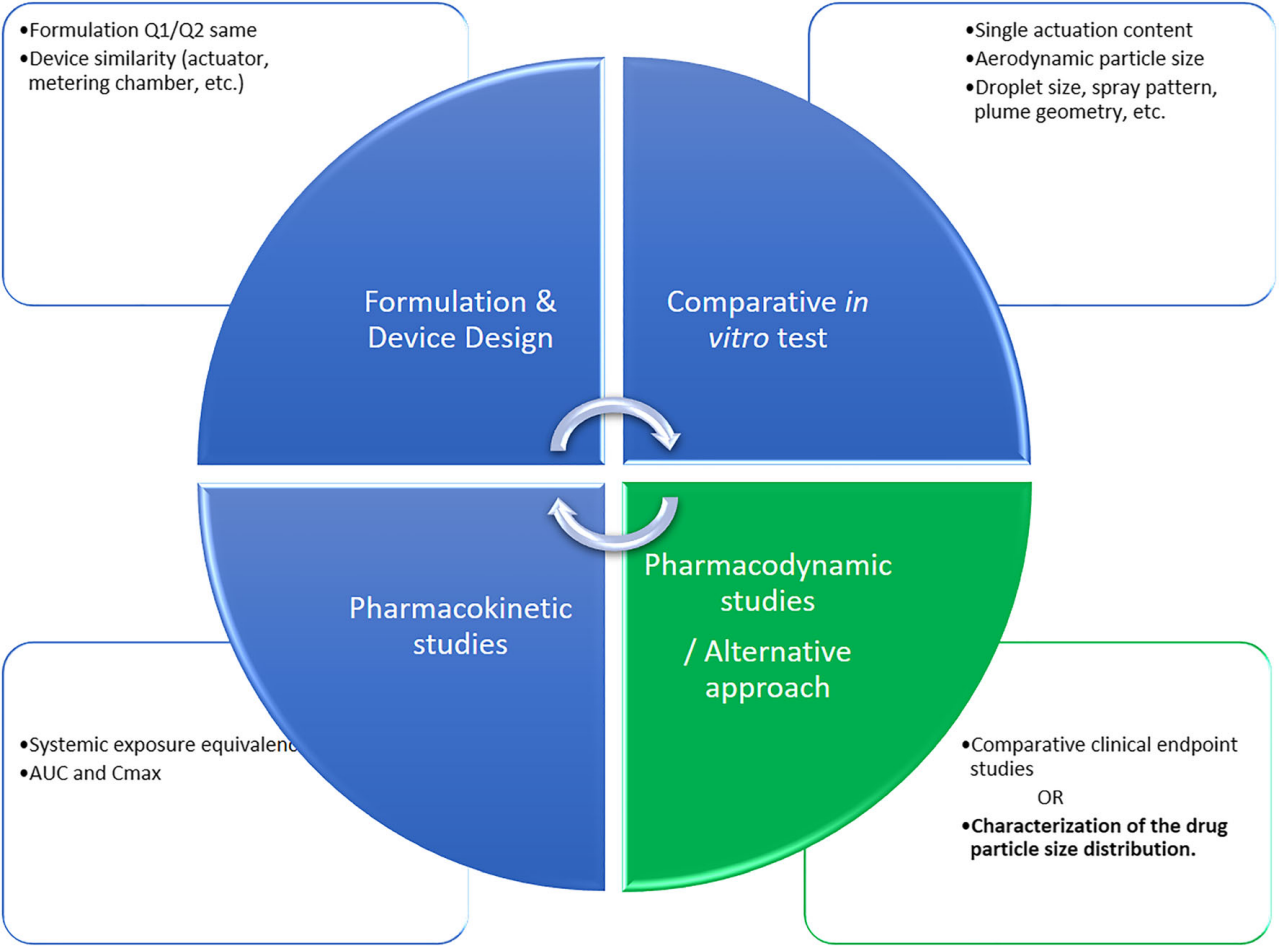
“Weight-of-evidence**”** approach for demonstrating bioequivalence of locally acting nasal suspensions [8]

For an abbreviated new drug application (ANDA) submitted under section 505(j) of the Federal Food, Drug, and Cosmetic Act, the FDA recommends a “weight-of-evidence” approach to help determine BE between test and reference products for locally acting nasal suspensions as presented in Fig. 1 [4, 6]. In line with the Generic Drug User Fee Amendments (GDUFA) program and the “weight-of-evidence” approach, the FDA has published a number of product-specific guidances (PSGs) for a series of nasal products [7, 8]. A common thread in all PSGs for locally acting nasal products is the recommendation that the test nasal product formulation is qualitatively (Q1) and quantitatively (Q2) the same as the nasal reference product in terms of inactive pharmaceutical ingredients. Current PSGs drafted by the FDA state the device should have a similar design and user interface with similar external operating principles and external critical design attributes, size and shape, and the number of doses [8]. A further recommendation is to perform *in vitro* studies to determine *in vitro* BE through single actuation content (SAC), droplet size distribution (DSD) by laser diffraction, drug in small particles by cascade impactor, spray pattern (SP), plume geometry (PG), and priming and repriming studies. *In vivo* pharmacokinetic (PK) studies to demonstrate BE in systemic exposure is also recommended. Since PK studies and current *in vitro* studies may not fully describe the fate of the drug in the nose with high resolution, demonstrating equivalence on local delivery should also be performed through comparative clinical endpoint BE studies [6, 9]. Although the “weight-of-evidence” approach comprises a robust strategy to demonstrate BE, the inclusion of a comparative clinical endpoint BE study can be a challenge to generic product development. Comparative clinical endpoint BE studies are expensive and can add US $2–6 million to the cost, are time-consuming, pose challenges with recruiting patients during allergic rhinitis season, and the results can be highly variable and unpredictable in many cases [10, 11].

Nasal suspension drug products consist of API particles suspended in an aqueous system in the presence of a range of different excipients. For nasal suspension products, the API particle size is a key critical material attribute which affects emitted API particle size and regional deposition of API in the nose [12–14]. In addition, the particle size of the API will affect the rate of dissolution and absorption at the site of deposition into the nasal epithelium and systemic circulation.

In March 2016, the FDA’s Office of Generic Drugs (OGD) approved a mometasone furoate nasal suspension generic drug product. The data supporting the application included using an *in vitro* tool called morphologically-directed Raman spectroscopy (MDRS) *in lieu* of a comparative clinical endpoint BE study [1]. In comparison to other Raman chemical imaging approaches for nasal sprays [13], MDRS measures morphological characteristics (size and shape) using its microscopic component to focus the analysis on drug particles and performs chemical identification by Raman spectra. This technology enables a comparison of the particle size of API in the generic and innovator drug products. Subsequently, the FDA has issued revised PSGs for locally acting nasal suspensions that include recommendations for an alternative BE approach utilizing the MDRS method (or any other similar advanced methodology) *in lieu* of comparative clinical endpoint studies [7, 8]. The publication of these PSGs is the result of efforts from the FDA in finding and developing novel techniques that can be validated and enhance the scientific evidence for BE studies without compromising the quality of the product [15–17].

Although particle size distribution (PSD) of the API can be readily determined by a number of methods prior to formulation and manufacture of the finished product, the primary challenge has been to determine the PSD of the API in the finished nasal aqueous suspension in the presence of undissolved excipients [13, 18]. Excipients such as microcrystalline cellulose typically have a median particle size that is larger than the API. However, such excipients often exhibit a broad PSD; thus, a substantial number of particles may exist in the same size range as the API itself, complicating the API particle size determination. MDRS measures particle morphological characteristics (size and shape) using its microscopic component and performs chemical identification by analyzing Raman spectra. The observed particles in a given sample can be classified based on morphology and/or Raman spectra. The selected particles are then characterized for size distribution using the microscopic technique. Hence, the MDRS method has been utilized for ingredient (API)-specific particle size measurement in a sample containing both API and excipient particles.

After intranasal application of the aqueous glucocorticoid suspension, the drug crystals have to dissolve in the epithelial mucous fluid layer. A sustained dissolution of drug particles contributes to prolonged nasal contact time [14, 18, 19]. Since dissolution of the drug substance is directly related to the particle size of the API, the measurement of dissolution of APIs in the nasal suspension formulation may be an orthogonal technique to the measurement of API particle size (i.e., differences in test and reference product dissolution can confirm similarities or differences in the API particle size in the formulation). Therefore, it is proposed that the API dissolution of nasal suspensions is a critical measurement that links to the API particle size in suspension. Moreover, measurement of the dissolution may help to validate the particle size tools for assessing size the drug particle substance in suspension.

The key objective of this study was to use a combination of techniques to investigate the drug substance particle size in nasal suspensions and dissolution rate to characterize test and reference nasal suspensions. In this study, MDRS and dissolution were evaluated for their discriminatory capability in the measurement of API particle size in nasal suspension formulations.

## MATERIALS AND METHODS

Four batches (Batch 1, 2, 3, and 4) of micronized mometasone furoate monohydrate (Sterling, Perugia, Italy) were procured and formulated into aqueous nasal suspension sprays. A commercial blend (Avicel ® RC-591) of microcrystalline cellulose (MCC) and carboxymethylcellulose (CMC) was supplied by FMC Biopolymer (Brussels, Belgium) and used as a suspending agent for the manufactured nasal suspensions. The formulation composition was designed to be similar to the RLD product, Nasonex® (Merck, USA), and after being formulated, these were filled into white HDPE bottles and fitted with a screw-on VP3 pump supplied by Aptar Pharma (18/415, Le Vaudreuil, France). Nasonex® was also sourced for the investigations (Lot No. 14MAA532A, expiry: 10/2016). Solvents and excipients were supplied by Fisher Scientific UK (Loughborough, UK) as high-performance liquid chromatography (HPLC) and reagent grade, respectively. Ultra-pure water was prepared by Milli-Q using reverse osmosis (Merck, Darmstadt, Germany).

### Particle Size Distribution (PSD) of As-Received Mometasone Furoate Monohydrate (MFM)

PSD analysis of the as-received API batches of MFM was performed using wet dispersion laser diffraction particle sizing (Spraytec® with a wet dispersion unit, Malvern Panalytical, Worcestershire, UK). The API was dispersed in 0.05% lecithin in cyclohexane with internal sonication for 1 min. This preparation technique has been shown previously to not result in fracturing of micronized materials [20]. A sample was then added into the wet dispersion cell until 4–12% obscuration was reached at 3000 rpm. The average PSD over a 15-s period was performed, and Mie theory was used to further analyze the data [21].

Automated imaging by Morphologi G3-ID® (Malvern Panalytical, Worcestershire, UK) was also used to measure the as-received MFM API PSD. The API was dispersed in 0.05% lecithin in cyclohexane with internal sonication for 1 min. A sample of 0.5 mL was then pipetted with a plastic Pasteur pipette and slowly dispersed onto a quartz slide with circular movements to ensure full coverage onto the microscope slide. A plastic lid was placed over the slide to cover it partially and allow the slow evaporation of the volatile solvent to prevent agglomeration of the API into the center of the slide.

Both techniques, the automated imaging by Morphologi G3-ID® and laser diffraction by Spraytec® with a wet dispersion unit, were used to assess the PSD by volume distribution of the raw API materials. Each experiment was performed in triplicate.

### Manufacture of Nasal Suspensions

Four batches of nasal sprays were prepared with different batches of raw MFM API to be Q1/Q2 similar to Nasonex® (7). An aqueous solution of the polysorbate 80 (0.01% w/w, Spectrum, UK) was prepared into which MFM (0.05% w/w, Batch 1, 2, 3, or 4, Sterling, Perugia, Italy) was dispersed [22]. In a separate mixing vessel, the Avicel (2.00% w/w, Avicel® RC-591, FMC Biopolymer, Brussels, Belgium) was dispersed in purified water by homogenization. These two suspensions were combined with continuous stirring. Other agents such as glycerin, benzalkonium chloride, sodium citrate dihydrate, and citric acid monohydrate were added to the formulation (Fisher Scientific UK, Loughborough, UK). All formulations were filled into white HDPE bottles and fitted with a screw-on VP3 pump (18/415, Aptar Pharma, France). Ten bottles per batch were manufactured. Ten bottles of a placebo suspension were also manufactured with the same procedure and all the excipients, except the API.

### Single Actuation Content (SAC) of the MFM Nasal Suspensions

SAC was performed after priming the device ten times before collecting an individual sample into a scintillation vial [23]. Each actuation was collected by manually actuating a nasal spray pump and recovering the actuated dose in 100 mL of diluent (32.5 acetonitrile:32.5 methanol:35 Milli-Q Water). Ten repetitions of each product were analyzed via a suitable HPLC method (described below).

### Droplet Size Distribution (DSD) Analysis of the MFM Nasal Suspensions

DSD was measured using a Spraytec® (Malvern Panalytical, Worcestershire, UK) equipped with a 300-mm lens. The nasal spray was manually actuated at 3 cm from the laser in a carefully defined position with an extraction hood on top to capture the spray and prevent fallback of droplets through the beam. The RT Sizer software was used to capture the droplet size data at a frequency of 2.5 kHz for 0.6 s after the transmission dropped below 98%, while capturing the 0.1 s before dropping to this value. The average of 10%, 50% (volume median), and 90% of the cumulative volume undersize (d_10_, d_50_, and d_90_, respectively) during the fully developed phase of the spray was analyzed. All determinations were performed in triplicate after ensuring that the device was properly primed and by the same analyst to prevent any bias resulting from the different manual actuation profiles.

### Spray Pattern (SP) and Plume Geometry (PG) Measurements of the MFM Nasal Suspensions

SP and PG were determined by using Oxford Laser’s Envision system. This system combines a laser sheet and high-speed camera specifically designed for the characterization of nasal sprays. While for SP, the laser sheet was positioned at 3 cm from the nasal pump nozzle tip, for PG analysis, the whole plume of the spray was captured. All actuations were actuated upward manually, and an extraction unit was positioned above the laser line to avoid fallback of droplets. Data were analyzed with Oxford Lasers EnVision Patternate software. The plume width and angle were characterized for PG analysis, and the SP area and ratio of maximum and minimum diameter (ovality ratio) were calculated on a single frame during the fully developed phase. All determinations were performed in triplicate by evaluating one actuation per repetition after ensuring that the device was properly primed and by the same analyst to prevent any bias resulting from the different manual actuation profiles.

### Dissolution Analysis of the MFM Nasal Suspensions

Dissolution analysis was performed on the manufactured complex nasal suspensions. These suspensions were actuated ten times into a scintillation vial, and a sample of 0.5 mL was pipetted into a dissolution vessel. All dissolution studies were conducted in a USP Apparatus II, also known as the Paddle Apparatus. All dissolution measurements were performed at 37°C in 600-mL pH 7.4 phosphate-buffered saline containing 2.0% w/v sodium dodecyl sulphate (SDS) dissolution medium with a stirring speed of 75 rpm in USP Apparatus II (Erweka GmbH, DT 126, Heusenstamm, Germany). For all dissolution experiments, 3-mL aliquots were withdrawn at 2.5, 5, 10, 15, 20, 25, 30, 60, 120, 180, and 240-min time intervals and filtered directly into HPLC vials. To maintain a constant volume in the dissolution vessel, the sampling volume was replaced with pre-warmed dissolution media. Each sample was analyzed on a suitable HPLC method (described below). The fractional percentage of the drug dissolved at each time point was determined by dividing the amount of drug by the total mass loading. Sink conditions were maintained during dissolution studies. The dissolution data reported here focused on MFM (the API) for all of the drug products analyzed. Each experiment was performed in triplicate.

The similarity between batches was assessed by evaluating the similarity factor, f_2_, of the average dissolution profile for the first 60 min as proposed for *in vitro* dissolution testing conducted by the current FDA guidance, *SUPAC-IR: Immediate-Release Solid Oral Dosage Forms: Scale-Up and Post-Approval Changes: Chemistry, Manufacturing and Controls, In Vitro Dissolution Testing, and In Vivo Bioequivalence Documentation* (November 1995) [24]. The dissolution half-life was also evaluated through a first-order kinetics model for the first 20 min to describe the dissolution profile of MFM [25].

### HPLC Analysis of MFM

Quantification of MFM utilized reversed phase HPLC method coupled with UV detection. The system consisted of an Agilent 1260 Infinity HPLC System comprising binary pump flowing at 2.0 mL/min through a Thermo Scientific ODS Hypersil, 150 × 4.6 mm 5-μm column, within a temperature-controlling column oven at 45°C and a UV detector set to 250 nm. The mobile phase consisted of a gradient of a buffered solution of sodium dihydrogen orthophosphate solution, pH 3.0, and HPLC grade acetonitrile at a proportion of 65:35 for 5.0 min, followed by a change of gradient to 33:67 until 5.5 min when it changed back to the original gradient for three more minutes.

### PSD of MFM by Morphologically-Directed Raman Spectroscopy (MDRS)

**Fig. 2:**
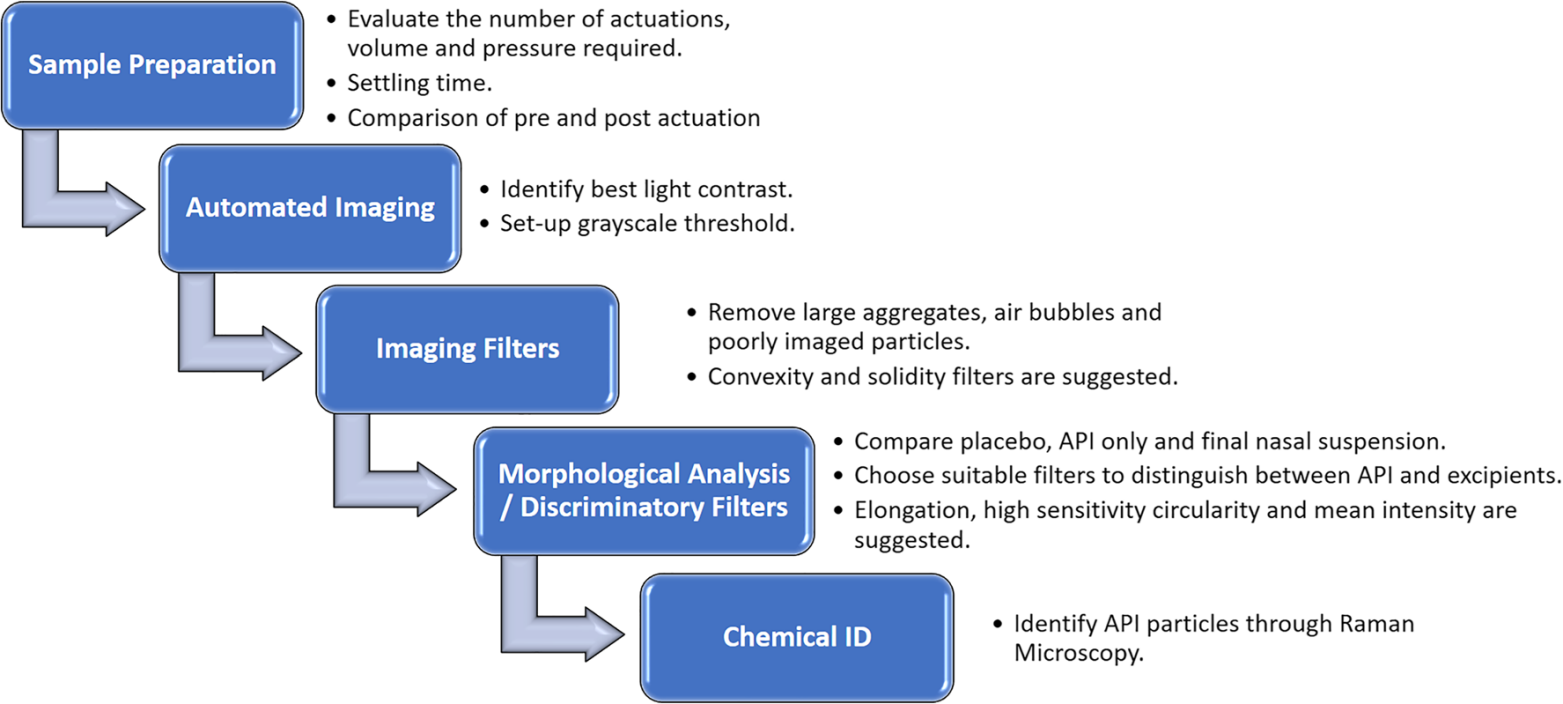
Process diagram of the method development stages for the particle sizing of MFM nasal suspension formulations

The morphology and particle size of the MFM API within the manufactured nasal suspension formulations was characterized using a Morphologi G3-ID morphologically-directed Raman spectroscopy system (Malvern Panalytical, Worcestershire, UK). The method development route for analysis of API in nasal formulations is shown in Fig. 2 and utilized the approach of optimizing sample preparation, imaging settings, applying imaging and API discriminatory morphological filters, and chemical analysis by Raman spectroscopy [26].

The method development started with the assessment of an optimized sample preparation method with Nasonex® where the number of actuations and distance from the nozzle to the scintillation vial necessary to have a repeatable homogenous sample, volume, and pressure required to spread the suspension below the coverslip and settling time and actuation effect on the PSD were investigated. During this evaluation, it was determined that five actuations with the nozzle of the nasal spray inside the scintillation vial provide representative and repeatable measurements of particle size of the nasal product. Furthermore, pipetting 3.3 μL onto a microscope slide without applying any pressure was able to spread a thin layer of the sample on the entire coverslip area with repeatable size measurements and minimum input from the analyst. Moreover, leaving the sample to rest for at least 60 min before the analysis was considered essential to allow the particles to settle until no movement is observed. The PSD of pre- and post-actuations of the sample (e.g., sample taken from bottle and sample actuated from the bottle) were found to be comparable. After optimizing the sample preparation method, a minimal amount of API-API or API-excipient agglomerates were noted for all batches, suggesting that this preparation method with shaking and actuation successfully dispersed any loose agglomerates [26].

During the microscopic measurement, the light settings and thresholds were defined in a Nasonex® sample to ensure good contrast between particles and background and to capture the whole perimeter of the particles being analyzed. A 50× magnification was used to capture the micron size particles. Then, morphological filters, such as convexity < 0.9, solidity < 0.9, and intensity standard deviation < 20.000, were used to remove poorly imaged particles and aggregates, as recommended by the FDA [26]. An intensity standard deviation between 35.000 and 80.000 and a solidity higher than 0.8 were used to exclude air bubbles in the sample for chemical analysis. Before the chemical analysis, a nasal formulation was compared with a placebo. The main goal of this comparison was to identify particle morphology filters that could be used to improve the targeting of API particles for chemical analysis. Figure S1 in supplementary data demonstrates that excipient particles (red boxes) in Nasonex® are more elongated than drug particles (blue circles). Applying a filter based on elongation percentage within a range of 0.3–0.5 increased the sampling of many thousands of API particles compared to the analysis without any filter which captures mostly excipient particles, as per Fig. S2 in supplementary data. An elongation filter of 0.3 was used.

Upon applying these filters, the chemical analysis was carried out using the Kaiser Optical Systems RamanRxn1 Spectrometer integrated with the Morphologi G3-ID equipment. The Raman spectrum for each of the particles of the same scanning area was collected using 60 s of exposure time with excitation at a wavelength of 785 nm over the spectral range of 100–1825 cm^−1^ at a resolution of 6 cm^−1^. After the chemical analysis, the collected spectra from each particle were compared against the reference spectra of MFM (Fig. S3 in supplementary data), and a correlation score was given to each particle. Particles with a score above 0.6 were classified as MFM. To facilitate the analysis of the collected spectra with minimum noise, only the spectra range between 1350 and 1750 cm^−1^ was used for correlation to the library spectra since the main identifiable peaks for MFM (1397 cm^−1^, 1471 cm^−1^,1660 cm^−1^, and 1708 cm^−1^) are within this range. Moreover, a background subtraction from an area of the analyzed sample with no particles scaled to the signal based on its similarity, followed by the application of Savitsky-Golay filtering over 31 points (intermediate smoothing) and a second derivative of the signal were applied to reduce the noise in the spectrum while preserving the underlying signal [27]. All determinations were performed in triplicate after ensuring that the device was primed. A minimum of 150 particles chemically identified as MFM was required per replicate.

### Statistical Analysis

Statistical analysis between the different populations was carried out using one-way analysis of variance (ANOVA) followed by Tukey’s post hoc analysis. All statistical analyses were performed using Minitab 17 software (Minitab, Coventry, UK). Probability values of <0.05 were considered as statistically significant.

## RESULTS AND DISCUSSION

The local rate and extent of absorption of an API delivered intranasally via a suspension nasal spray are related to the size of the drug crystals in the suspension. The particle size of the API will govern the dissolution rate of the drug crystals and, therefore, absorption of the drug locally in the nasal cavity. Hence, measurement of the particle size of the API *in situ* within the nasal spray suspension would provide relevant data that would help predict the local rate and extent of absorption of the API within the nose. With the advent of the MDRS approach to measure the particle size of APIs *in situ* within locally acting nasal suspension drug products, it is important to determine if the technique is able to track the particle size of the API pre- and post-manufacture of a locally acting suspension nasal spray. This is vitally important to ensure the technique is able to discriminate between the particle size of the suspended APIs from those of the undissolved excipients in the nasal suspension. In addition, it will be helpful to determine if particle size differences determined by MDRS are likely to result in similar trends seen in the dissolution kinetics.

### Particle Sizing of As-Received Mometasone Furoate Monohydrate (MFM)

**Fig. 3:**
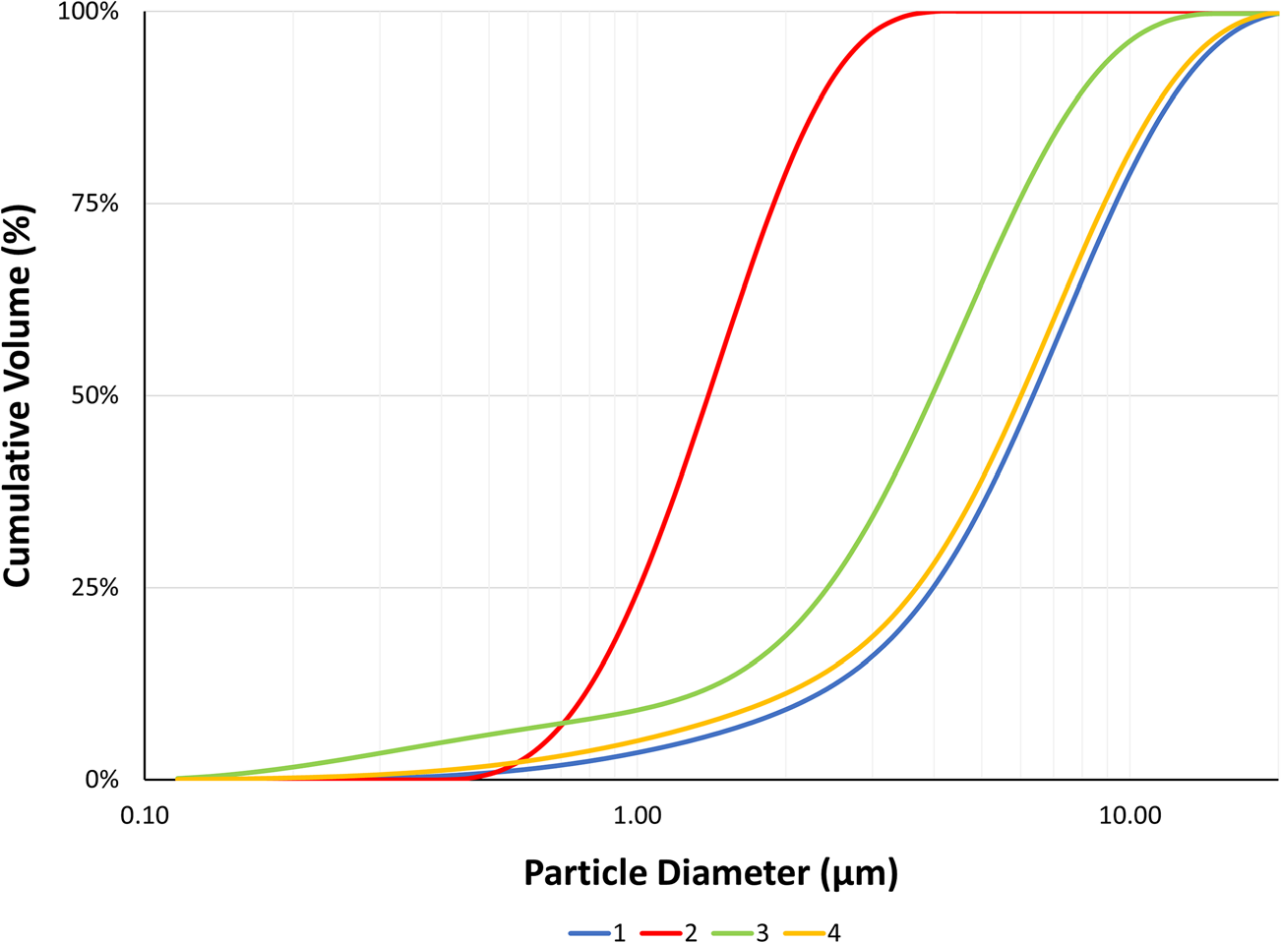
Mean PSD in the volume distribution of four batches of as-received MFM by laser diffraction (*n* = 3)

Four batches of MFM API were procured and sized before being formulated into nasal suspension formulations. The PSD measured by laser diffraction of the as-received MFM API Batches 1, 2, 3, and 4 are shown in Table I and Fig. 3. ANOVA followed by Tukey’s post hoc analysis was conducted on these data. All batches had a significantly different d_50_ (*p* << 0.05), and these data show that Batch 2 was significantly smaller than all other batches followed by Batch 3, Batch 4, and Batch 1. Furthermore, the Span was also significantly different (*p* << 0.05) between all batches.

**Table I:** The Mean PSD in the Volume Distribution of Four Batches of As-Received MFM by Laser Diffraction and Automated Imaging and After Being Formulated into Nasal Suspensions by MDRS in Comparison to a Nasonex***®*** Batch. Standard Deviations Are Included in the Parenthesis (***n*** = 3)

Technique	Batch	d_10_ (μm)	d_50_ (μm)	d_90_ (μm)	Span
Laser diffraction (as-received)	1	2.14 (0.05)	6.36 (0.08)	12.57 (0.11)	1.64 (0.01)
	2	0.76 (0.01)	1.39 (0.01)	2.42 (0.03)	1.19 (0.01)
	3	1.14 (0.01)	3.97 (0.02)	8.11 (0.10)	1.76 (0.02)
	4	1.81 (0.05)	6.01 (0.15)	11.94 (0.25)	1.69 (0.01)
Automated imaging (as-received)	1	2.81 (0.05)	6.84 (0.50)	10.09 (0.48)	1.07 (0.02)
	2	1.63 (0.19)	2.54 (0.24)	3.77 (0.34)	0.84 (0.08)
	3	3.69 (0.15)	5.80 (0.04)	8.14 (0.26)	0.77 (0.02)
	4	2.60 (1.13)	6.54 (0.23)	9.72 (0.20)	1.09 (0.23)
MDRS (final product)	1	2.72 (0.29)	5.64 (0.62)	10.26 (1.36)	1.36 (0.43)
	2	2.05 (0.01)	2.43 (0.03)	3.41 (0.15)	0.56 (0.06)
	3	2.47 (0.20)	4.21 (0.46)	6.60 (0.40)	0.98 (0.06)
	4	2.30 (0.01)	4.03 (0.04)	6.33 (0.07)	1.00 (0.01)
	Nasonex®	2.28 (0.14)	3.20 (0.92)	5.47 (1.28)	0.98 (0.14)

*PSD* particle size distribution, *MFM* mometasone furoate monohydrate, *MDRS* morphologically-directed Raman spectroscopy

**Fig. 4:**
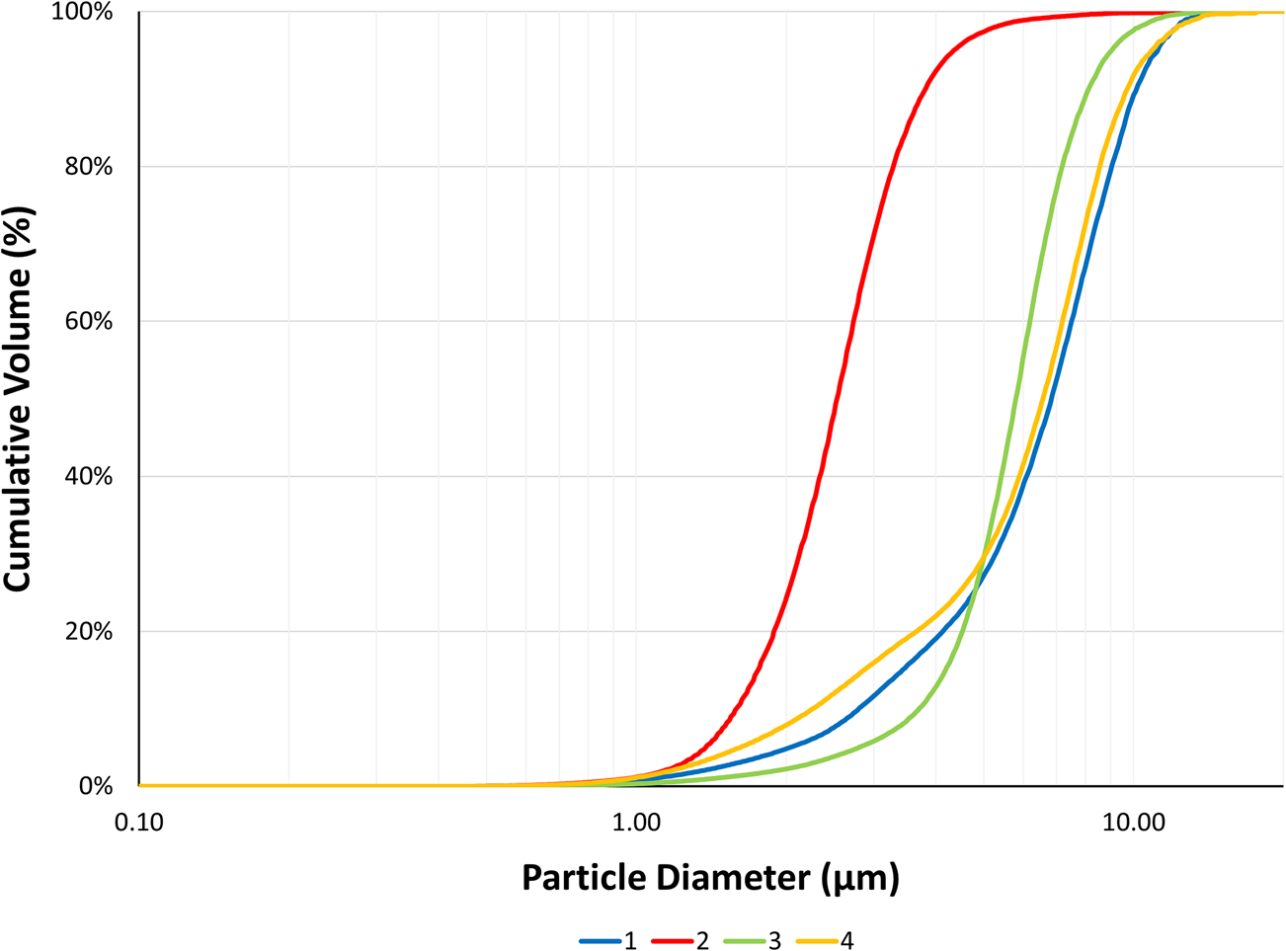
Mean PSD in the volume distribution of four batches of as-received MFM by automated imaging (*n* = 3)

The PSDs of the different API batches were also analyzed with automated imaging, which is the sizing methodology used by MDRS, to account for any differences between the between laser diffraction and MDRS techniques. To be able to compare these data from automated imaging and by MDRS with laser diffraction results, a conversion from number to volume distribution was required. These data are presented in Table I and Fig. 4 and suggest that the same trends as laser diffraction are observed for the as-received material. However, a larger PSD was observed when comparing the results obtained using laser diffraction. This difference is largely attributed to the sizing methods used for each technique. For example, automated imaging is an image-based particle sizing tool, while laser diffraction relies on diffraction of laser light to determine the particle size. Both techniques have a different limit of detection, which corresponds to 0.1 and 0.5 μm for laser diffraction and automated imaging, respectively [28, 29]. Also, the particle size data from laser diffraction-based methods use an equivalent sphere fit model which may explain the differences in PSD between automated imaging and laser diffraction. Additionally, while laser diffraction analyzes millions of particles with an algorithm that evaluates the data in volume distribution, automated imaging relies on the analysis of a limited number of particles (a few hundred) by counting and measuring every single particle individually and gathering the data as number distribution. Although volume distribution is generally preferred in the pharmaceutical industry due to its sensitivity to small changes in the amount of large material in the sample, in a conversion of number to volume distribution the error of the measurement will be cubed [18, 29, 30].

Despite these differences between techniques, there was generally good agreement between the rank order of particle size of the different batches captured by automated imaging, which followed the order Batch 1~Batch 4>Batch 3>Batch 2 for the as-received API. Hence, Batch 1 had the largest particle size (d_50_), which was not significantly different from Batch 4 but was significantly different (*p* < 0.05) from the other batches. Batch 2 had the smallest median diameter significantly different (*p* < 0.05) from all other batches. Batches 3 and 4 d_50_ were not statistically different. No statistical differences were observed for Span between the different batches, with the exception of Batch 4 and Batch 3 (*p* < 0.05).

Although a similar trend is observed in data captured by automated imaging when compared to the laser diffraction data, unlike laser diffraction, the differences in size between some batches do not appear to be significantly different, suggesting that laser diffraction is a more sensitive technique for measurement of PSD on the raw material than automated imaging. However, laser diffraction does not allow the measurement of the drug substance PSD of the finished product *in situ*. Therefore, it is critical to compare automated imaging results via MDRS with a more sensitive technique such as laser diffraction to ensure that both techniques are providing comparable results but also to measure the API PSD using MDRS prior to and after manufacturing the nasal suspension formulation batches to track any changes in the API PSD caused by the manufacturing process. Moreover, when comparing data from the same instrument, it is recommended to avoid the number-volume distribution conversion [29, 31].

### Manufacturing of Nasal Formulations and *In Vitro* BE Testing

In this study, four different particle size fractions of the API MFM were procured and formulated into four nasal suspension formulations (Batch 1, 2, 3, and 4) to be Q1 and Q2 the same to Nasonex®. These final batches were then submitted to most of the *in vitro* BE tests recommended in the PSG for *Mometasone Furoate Nasal Spray, Metered* (Recommended Sep 2015; Revised Feb 2019, Jun 2020): SAC, DSD by laser diffraction, SP, and PG [7, 8]. There were no statistical differences for SAC, DSD d_50_ and Span, SP ovality ratio or area, and PG width and angle of these formulations, as presented in Table II. Therefore, the only parameter that was purposely different between the formulated MFM nasal suspension products and Nasonex® was the particle sizes of the APIs included in the four test formulations, which did not appear to significantly impact these *in vitro* characteristics measured. This may suggest that the API particle sizes chosen in these manufactured batches were not impacted by the device design in these cases (i.e., formulation-device interactions) to achieve any significant differences upon actuation. In addition, this suggests an additional technique, such as MDRS, is necessary to characterize PSD of the API once formulated.

**Table II:** In Vitro Characterization of Four Batches of Formulated MFM and Nasonex® by Single Actuation Content (SAC), Droplet Size Distribution (DSD), Spray Pattern (SP), and Plume Geometry (PG). Mean Values and Standard Deviations in the Parenthesis are Presented (*n* = 3)

Batch	SAC (μg)	DSD d_50_ (μm)	DSD Span	SP ovality ratio	SP area (cm^2^)	Plume width (cm)	Plume angle (°)
1	49.45 (0.62)	42.17 (2.26)	1.37 (0.07)	1.45 (0.09)	4.31 (0.80)	4.26 (0.35)	41.39 (2.97)
2	49.94 (0.64)	40.34 (0.69)	1.42 (0.03)	1.44 (0.10)	4.15 (0.91)	4.56 (1.29)	40.70 (9.96)
3	49.88 (0.45)	40.06 (1.16)	1.42 (0.06)	1.37 (0.11)	4.81 (0.55)	4.33 (0.85)	41.24 (8.61)
4	49.59 (0.91)	42.01 (1.72)	1.43 (0.02)	1.39 (0.08)	4.66 (0.79)	4.21 (0.92)	39.77 (10.01)
Nasonex®	49.49 (0.79)	42.8 (0.46)	1.43 (0.02)	1.84 (0.54)	4.38 (0.44)	4.61 (0.39)	40.74 (2.35)

*MFM* mometasone furoate monohydrate

### *In Situ* Particle Sizing and Morphology Analysis of Nasal Suspensions Using Morphologi G3-ID

**Fig. 5:**
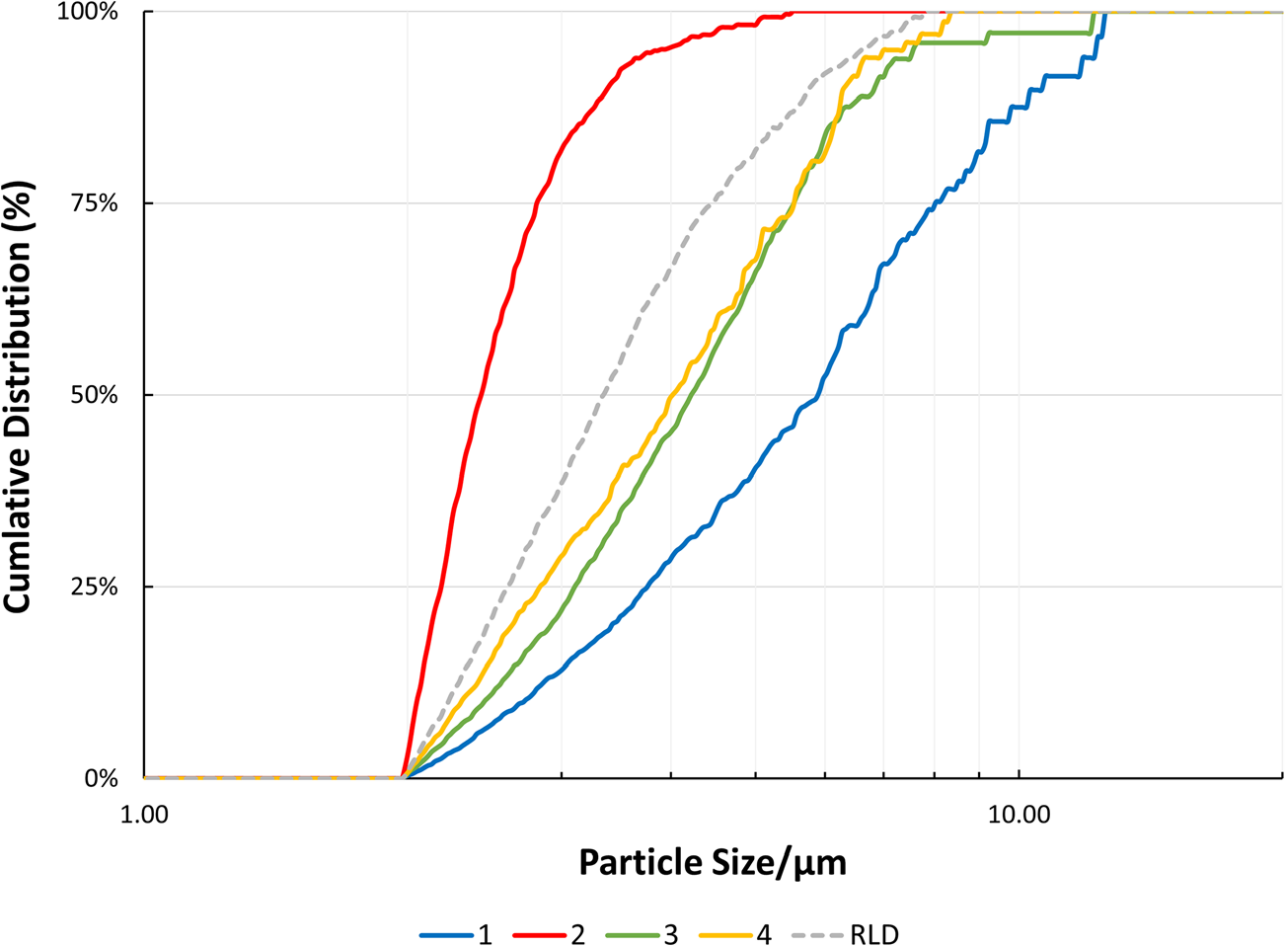
Mean MDRS PSD in the volume distribution of four batches of MFM API formulated into aqueous nasal suspension formulations and Nasonex® (*n* = 3)

The API batches were manufactured as aqueous nasal suspensions to be similar to Nasonex® but formulated with API batches with different particle sizes. The MDRS method was then employed to determine if the as-received drug substance particle size correlated with the formulated drug substance particle size in the formulation and released from the nasal spray device. These data are presented in Table I and Fig. 5.

Generally, the API particle size in the formulated products appeared to have less fines (d_10_) than the as-received API when measured by laser diffraction. Besides the previously discussed differences between the two techniques, this difference may also indicate that the API may have undergone Ostwald ripening in the aqueous vehicle [32–34]. When comparing MDRS on the final product with a closer methodology (automated imaging), a general API particle size reduction was observed for all batches (Table I), with Batch 3 and Batch 4 showing statistical differences before and after formulation (*p* < 0.01). Even though these batches were formulated under the same conditions, this reduction in API particle size was particularly more pronounced for Batch 4 and can be associated with the higher friability of this API batch towards the high shear homogenization [35, 36]. Although this reduction in particle size could also be associated with the forces involved during the formulation actuation from a nasal spray pump through a small nozzle orifice, there was no evidence of any actuation effect observed during the MDRS method development.

Moreover, these data show that Batch 2 d_50_ was significantly (*p* < 0.05) smaller than all other test batches in the manufactured formulations (Table I) as observed by laser diffraction (Table I) and automated imaging analysis of the raw API (Table I). Batch 1 has a significantly (*p* < 0.05) larger d_50_ when comparing to any other formulated batch. Considering the formulated products, the median size for Batch 4 was significantly smaller (*p* < 0.05) than Batch 1 (Table I) unlike what was observed for the as-received API material by automated imaging (Table I), where no statistical difference between these batches was found. The finished product d_50_ was not statistically different between Batch 3 and Batch 4 (Table I). Nevertheless, there was a good agreement in the rank order of particle sizes between the API raw material and API in the finished product, except for Batch 4 which might have undergone through a more pronounced particle size reduction during formulation. For the finished product, the following rank order of median particle size was observed: Batch 1>Batch 3~Batch 4>Batch 2. Only Batch 1 and Batch 2 were assessed as having a statistically different (*p* < 0.05) Span in comparison with each other. When comparing the test batches with Nasonex®, no significant difference was observed between Batch 2, 3, 4, and the Nasonex® for both d_50_ and Span.

### Dissolution as an Orthogonal Technique to Support MDRS

MDRS was utilized to track the PSDs of the API prior to and once incorporated into a complex nasal product. However, MDRS is an optical microscopy technique with limitations to the lowest detectable particle size (between 0.5 and 2 μm) [28]. Therefore, an orthogonal technique that can trace any difference in particle size of the API is required. Although various attempts have been made in the literature to model the PSD of the API from a dissolution profile based on the Noyes-Whitney equation, there is no universal approach for this prediction [37–42]. Nevertheless, dissolution analysis is a measure of surface area and is still a valid technique to track differences in PSD of hydrophobic drug substances in which dissolution is the rate-limiting step involved in the drug release into the media. In fact, dissolution is more sensitive to particles with higher surface area and smaller particle size, making this tool an ideal orthogonal technique to evaluate any differences in the drug substance particle sizes. Other product attributes such as rheology and surface tension might also affect the release rate of the active ingredient particularly for more hydrophilic drug products and depending on the dissolution or *in vitro* release testing setup [3, 43, 44].

**Fig. 6:**
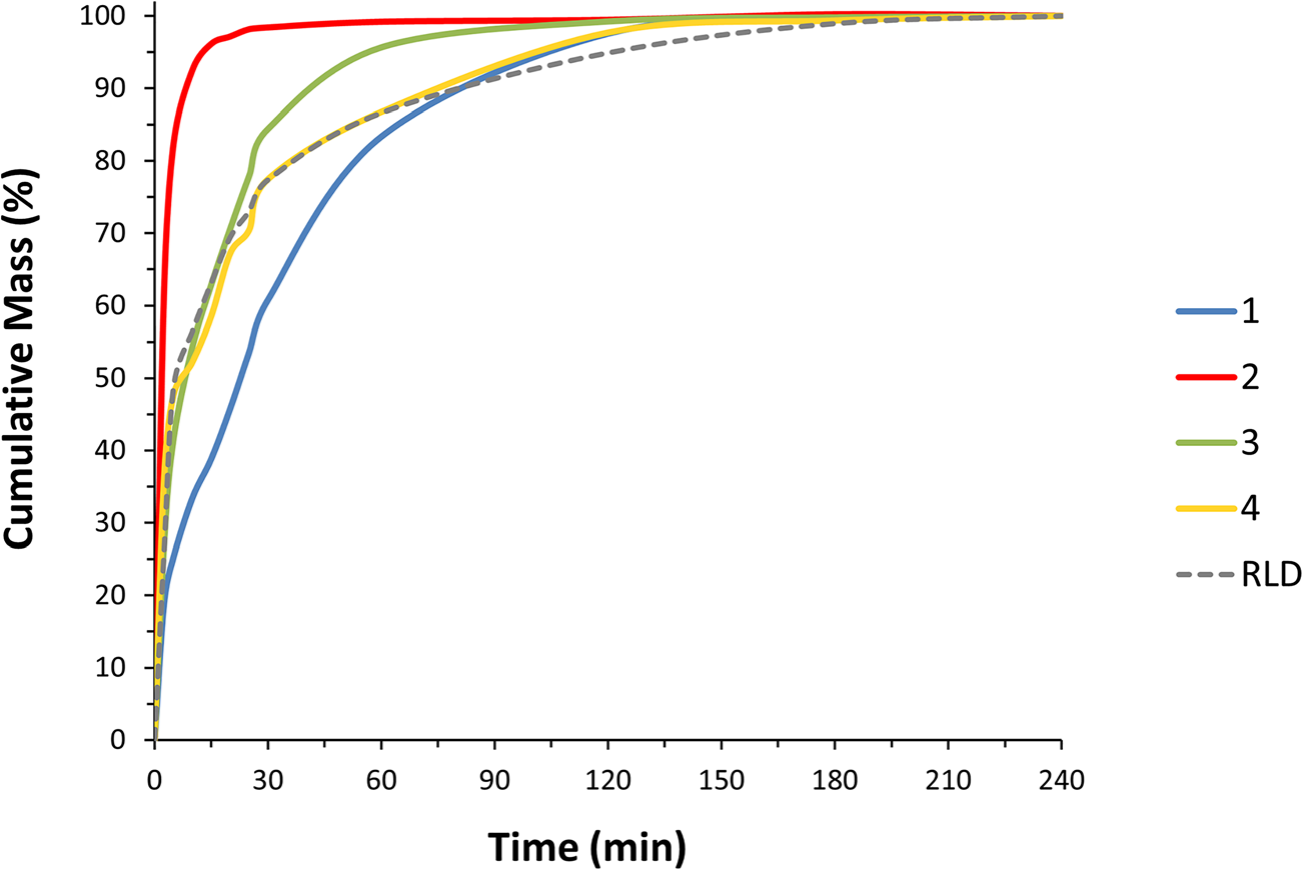
Mean dissolution profile (*n* = 3) of four batches of MFM API formulated into aqueous nasal suspension formulations and Nasonex®

The dissolution analysis of the formulations made with drug substance of different particle sizes was performed, and the results are presented in Fig. 6. The similarity between batches was assessed by evaluating the similarity factor f_2_ analysis, which is presented in Table III. These data suggest that Batch 3, Batch 4, and Nasonex® have a similar dissolution profile which correlates well with PSD data measured by means of MDRS, thus, supporting the previously observed reduction of API particle size for Batch 4 during formulation to a similar PSD as that of Batch 3 and Nasonex®. Furthermore, Batch 1 had the slowest dissolution rate correlating well with the largest API PSD, and Batch 2 the fastest dissolution rate with a strong correlation to the smallest API PSD.

**Table III:** Similarity Factor f_2_ Analysis of the Mean Dissolution Profile (*n* = 3) of Four Batches of MFM API Formulated into Aqueous Nasal Suspension Formulations and Nasonex®

Batch	2	3	4	Nasonex®
1	10.98	29.59	32.16	30.72
2	-	21.71	21.10	21.63
3	-	-	54.12	59.03
4	-	-	-	62.36

*MFM* mometasone furoate monohydrate, *API* active pharmaceutical ingredient

**Fig. 7:**
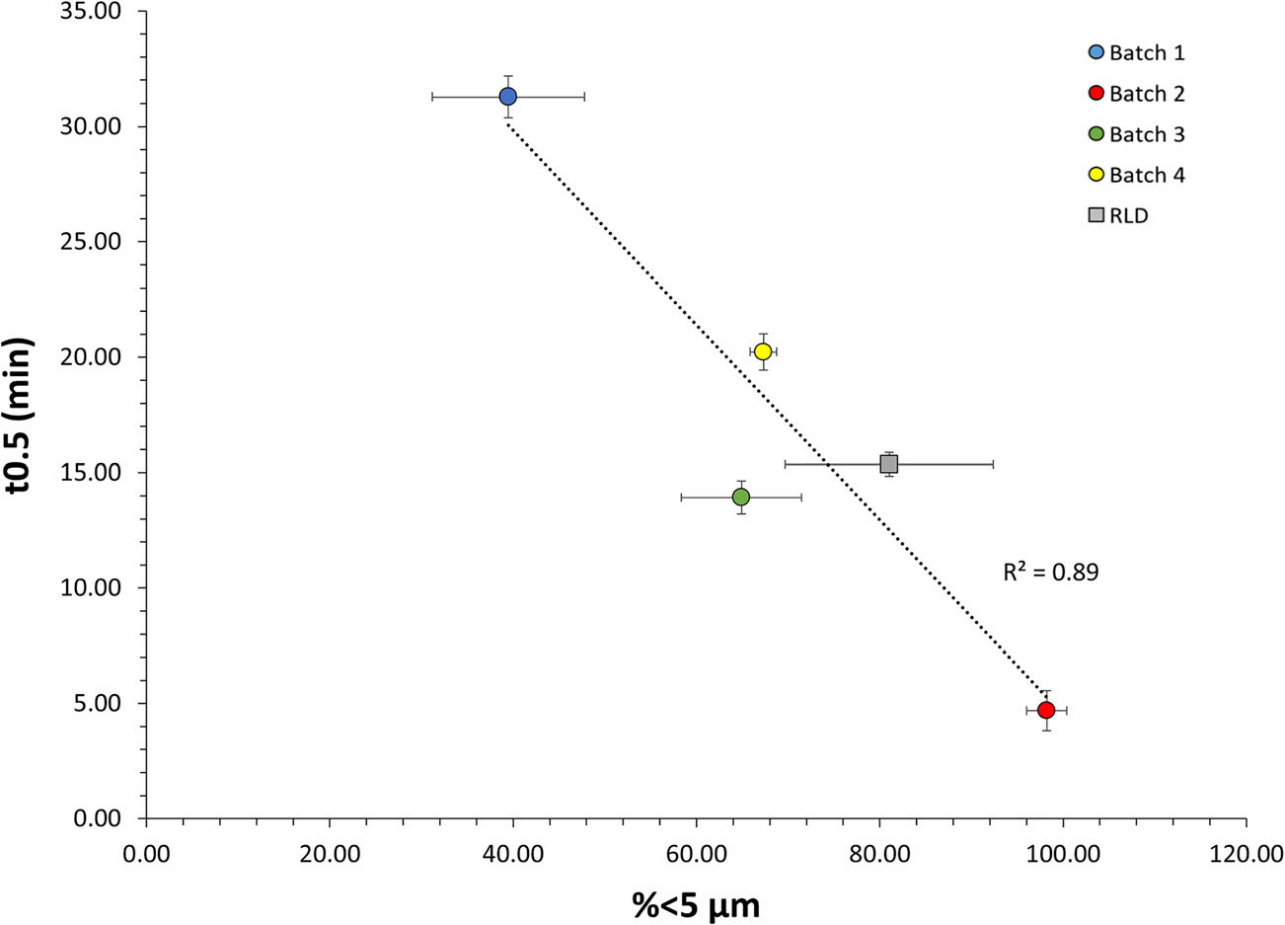
Relationship between the mean percentage by volume less than 5 μm (% < 5 μm) of the formulated MFM drug substance measured by MDRS and the mean dissolution half-life (t_0.5_) of the MFM drug product. Error bars show standard deviations (*n* = 3)

Since the finer particles will have the greatest effect on dissolution rate, the percentage by volume less than 5 μm (%<5 μm) (Table I) was compared against the dissolution half-life (T_0.5_) as presented in Fig. 7. These data suggested a good correlation between the %<5 μm and T_0.5_ of the formulated products for a limited number of batches analyzed. Hence, an orthogonal approach combining MDRS and dissolution analysis may be supportive for generic manufacturers in developing generic products of aqueous nasal suspensions and ensuring they have control of the drug product quality and BE. These experimental data, particularly for poorly soluble drugs, may also be used in combination with regional deposition results obtained via realistic nasal casts to feed an *in silico* model able to characterize the regional deposition, mucociliary clearance, and absorption that determine both local and systemic exposure [45–49]. Moreover, the combination of these orthogonal techniques may be used for BE studies as part of the alternative approach to the comparative clinical endpoint BE study proposed in the draft PSG for *Mometasone Furoate Nasal Spray, Metered* (Recommended Sep 2015; Revised Feb 2019, Jun 2020) [8]. This guidance was reissued in 2019 after a novel technology (MDRS) that was able to measure the API particle size within a complex nasal suspension emerged. This guidance reinforces the previous FDA approval of Apotex’s ANDA application for a generic copy of Merck’s Nasonex where the *in vitro* particle size data from MDRS was accepted *in lieu* of the comparative clinical endpoint BE study [1, 26]. The data presented herein suggest that MDRS allows the comparison of the particle size distribution of an API within a complex nasal suspension test and reference product by tracking the particle size before and after formulation and taking into account any changes during manufacturing and storage. Furthermore, orthogonal techniques such as dissolution may be used to strengthen the MDRS data by compensating for its limitations and evaluating differences in the dissolution rate of the drug substance particles.

## CONCLUSION

A combination of morphological analysis and Raman spectroscopy of a nasal suspension was used to isolate the API population for drug-specific particle sizing in formulated locally acting nasal suspension sprays. This approach allowed characterization of the drug substance PSDs in the formulation and thereby facilitates comparative analysis of test and reference products. Hence, these data suggest that MDRS may be used to evaluate the PSD of the API in a complex nasal suspension. Still, MDRS is a microscopy technique that has a limitation on the minimum particle size detected. To compensate for this limitation, the application of an orthogonal technique that is able to evaluate differences in API particle size is suggested. Herein, dissolution was successfully used as an orthogonal technique to track the API PSD of a complex nasal suspension. Hence, together these analytical methods may facilitate the determination of critical material and process attributes that may affect drug product quality and may aid in the assessment of BE determination between test and reference formulations.

## Supplementary Information

Fig. S1 Image of a Nasonex® nasal spray sample with some Avicel particles selected with red boxes and API particles selected with blue circles (PNG 288 kb)

Fig. S2 Comparison of the elongation distribution graph of Placebo (red) and Nasonex*®* (green) of a combined analysis of three repetitions (PNG 70 kb)

Fig. S3 Reference Raman spectra of MFM with the masking range between 1350 and 1750 cm^−1^ selected in white (PNG 30 kb)
