# Supplementary figures and images for: A Systematic Approach in the Development of the Morphologically-Directed Raman Spectroscopy Methodology for Characterizing Nasal Suspension Drug Products
Source: AAPS J. 2021 May 18;23(4):73. doi: 10.1208/s12248-021-00605-w (PMC8131332; doi:10.1208/s12248-021-00605-w)

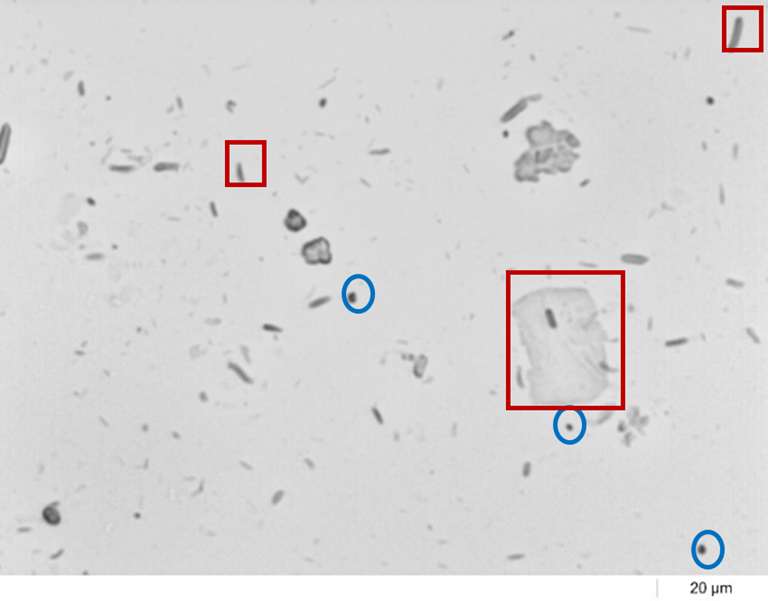

Supplement: Supplementary file 1 — Image of a Nasonex® nasal spray sample with some Avicel particles selected with red boxes and API particles selected with blue circles (PNG 288 kb) [file 12248_2021_605_MOESM1_ESM.png]

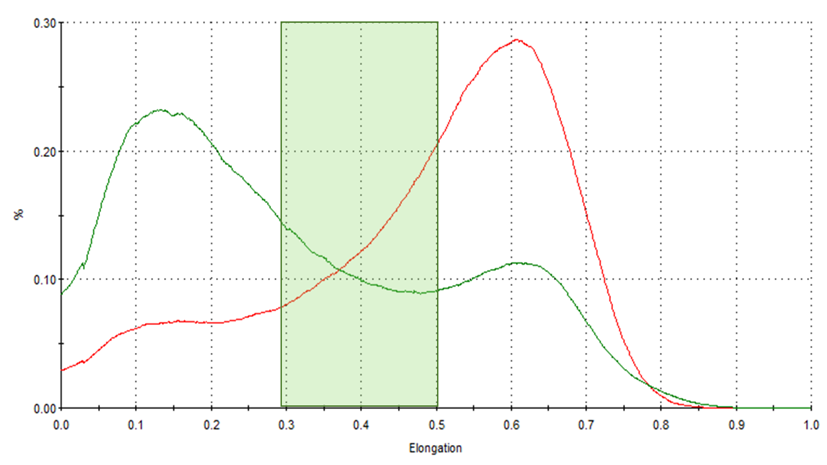

Supplement: Supplementary file 2 — Comparison of the elongation distribution graph of Placebo (red) and Nasonex® (green) of a combined analysis of three repetitions (PNG 70 kb) [file 12248_2021_605_MOESM2_ESM.png]

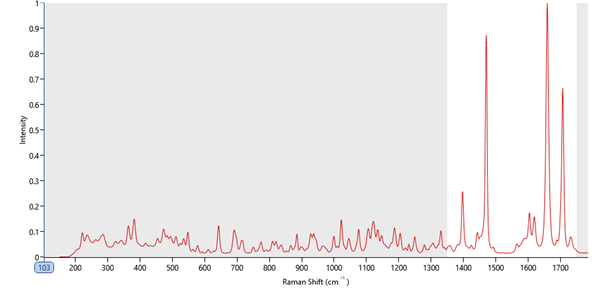

Supplement: Supplementary file 3 — Reference Raman spectra of MFM with the masking range between 1350 and 1750 cm−1 selected in white (PNG 30 kb) [file 12248_2021_605_MOESM3_ESM.png]
